# The effectiveness of pregabalin with or without agomelatine in the treatment of chronic low back pain: a double-blind, placebo-controlled, randomized clinical trial

**DOI:** 10.1186/s40360-022-00612-3

**Published:** 2022-09-14

**Authors:** Seyed Mani Mahdavi, Behnam Shariati, Mohammadreza Shalbafan, Vahid Rashedi, Masoomeh Yarahmadi, Alireza Ghaznavi, Shayan Amiri

**Affiliations:** 1 Bone and Joint Reconstruction Research Center, Department of Orthopedics, School of Medicine, Iran University of Medical Sciences, Tehran, Iran; 2 Mental Health Research Center, Department of Psychiatry, School of Medicine, Psychosocial Health Research Institute (PHRI), Iran University of Medical Sciences, Tehran, Iran; 3 Iranian Research Center on Aging, Department of Aging, University of Social Welfare and Rehabilitation Sciences, Tehran, Iran; 4 School of Behavioral Sciences and Mental Health, Iran University of Medical Sciences, Tehran, Iran

**Keywords:** Low back pain, Agomelatin, Pregabalin, Clinical trial

## Abstract

**Background:**

Although various pharmacological and nonpharmacological treatments are available for the chronic low back pain (CLBP), there is no consensus on the best optimal treatment for this condition. This study aimed to investigate the efficacy of co-administration of pregabalin and agomelatine versus pregabalin with placebo to treat CLBP.

**Methods:**

Forty-six CLBP patients without the surgical indication referred to the outpatient orthopedic clinic of Rasoul-e-Akram Hospital, Tehran, Iran, were randomly divided into two study groups: Group A [pregabalin (75 mg twice per day) + placebo] and Group B [pregabalin (75 mg twice per day) + agomelatine (25 mg per night)]. Patients were evaluated at weeks 0, 4, and 8. Outcome measures were the Persian versions of the Brief Pain Inventory (BPI) interference scale, Roland-Morris Disability Questionnaire (RMDQ), The Hospital Anxiety and Depression Scale (HADS), 36-Item Short Form Survey (SF-36), and General Health Questionnaire-28 (GHQ-28) were used.

**Results:**

At weeks 4 and 8 after the intervention, all evaluated measures showed significant improvement in both study groups (P < 0.01). The mean improvement of GHQ-28 was 3.7 ± 1.22 in group A and 13.1 ± 4.71 in group B. This difference was statistically significant (P = 0.003). Other outcomes did not vary substantially between the two research groups. Agomelatine treatment was well tolerated, with no significant adverse effects seen in patients. Liver tests of all patients were routine during the study period. Major adverse effect was not seen in any patient. The prevalence of Minor side effects was not significantly different between two study groups.

**Conclusion:**

Compared with the pregabalin and placebo, co-administration of pregabalin and agomelatine had no added effect on improving pain scores in CLBP patients. However, the patients’ general health was significantly improved after the combined administration of pregabalin and agomelatine.

**Trial registration:**

The study protocol was registered in the Iranian Registry of Clinical Trials before starting the study (NO.IRCT20200620047852N1, Registration date: 23/06/2020).

## Background

Chronic low back pain (CLBP) is referred as back pain secondary to various mechanical and nonmechanical disorders lasting for more than three months [1]. CLBP is a widespread health problem affecting 50–80% of the population at some point in life [2]. The prevalence of CLBP is almost 4.2% at the age of 24–39 years and 19.6% at the age of 20–59 years [3]. Years lived with disability (YLDs) of low back pain (LBP) has increased by 52.7% from 1990 to 2017 [4]. It is connected with significant impairment, treatment expenses, and sick leave, in addition to being the top reason of seeking health care services [5]. As a result, discovering effective CLBP treatment techniques is crucial.Although numerous pharmacologic and non-pharmacologic modalities of treatments are available for CLBP, its treatment remains a clinical challenge for physicians [6]. One of the underlying factors limiting the success of CLBP treatment is inattention to the pain mechanism. Chronic low back pain is a complex and heterogeneous condition in which both the nociceptive and neuropathic pain mechanisms could be involved.

The neuropathic component of pain is present in 16 to 55% of CLBP patients [7, 8]. The average cost of annual care for each CLBP patient with a neuropathic component is approximately 160% higher than for patients without neuropathic pain [9]. As a result, distinguishing between the nociceptive and neuropathic mechanisms of CLBP, as well as mechanism-based treatment of this illness, is clinically important for improving patient outcomes and lowering disease burden.However, in most cases, CLBP is treated regardless of the pain mechanism, and present treatment instructions usually do not provide specific recommendations to treat the neuropathic components of CLBP [8].

Pregabalin is an alkylated analog of gamma-Aminobutyric acid structurally similar to gabapentin. It contains anticonvulsant, analgesic, and anti-anxiety effects [7] which is commonly used to treat neuropathic CLBP [10]. However, many patients report no beneficial results for pregabalin and discontinue treatment [10, 11]. Therefore, efforts continue to develop more effective pharmacologic therapies for the neuropathic component of pain in CLBP patients.

Agomelatine is a new class of antidepressant drugs which act as an MT1 and MT2 melatonergic receptor agonist and a 5-HT receptor antagonist. Agomelatine’s antagonistic impact causes the limbic system, which is implicated in the pathophysiology of neuropathic pain, to produce more norepinephrine [12].In a recent study by Chenaf et al., agomelatine administration in an experimental model reduced neuropathic pain, and this effect was intensified if agomelatine was co-administered with gabapentin [13]. However, the effect of agomelatine as an adjunct treatment in the human CLBP was not investigated in any earlier studies.

We aim to evaluate the therapeutic effects of pregabalin and agomelatine co-administration versus pregabalin alone to determine whether the combination of these agents will result in more effective treatment of CLBP.

## Patients & methods

### Study design

Institutional Review Board of Iran University of Medical Sciences approved this study under the code IR.IUMS.FMD.REC.1399.200. Written informed consent was taken from the patients before participating in the study. The study protocol was registered in the Iranian Registry of Clinical Trials prior starting the study (NO.IRCT20200620047852N1, registration date: 23/06/2020).

In a double-blind, randomized, placebo-controlled trial, 46 CLBP patients referred to the outpatient orthopedic clinic of Rasoul-e-Akram Hospital, Tehran, Iran, were included. The inclusion criteria were 18–60 years, CLBP lasting for at least six months, and no indication for surgery. Patients were not included if they were used opioid, antidepressant, benzodiazepine, and gabaergic medications for an extended period, patients with contraindication for pregabalin or agomelatine administration, patients using CYP1A2 inhibitors such as fluvoxamine or ciprofloxacin, patients with conditions interfering with the therapeutic results, such as life expectancy of fewer than 12 months, patients with liver enzyme three times higher than usual, patients with severe depression, suicidal ideation, psychosis, acute phase of mania, severe cognitive impairment, anxiety disorder after severe trauma, and any psychiatric disorder requiring standard pharmacological treatment beside the study treatment, patients who were under treatment with pregabalin or any other antidepressants for the past month, and patients undergoing physiotherapy. No expectant or nursing moms participated in the trial either. Consent withdrawal, severe pharmacological adverse effects such as rise of liver enzyme to three times the typical value in the third week, change in treatment method for any reason and needing surgical intervention, and pregnancy during treatment were all disqualifying factors.

### Randomization

Participants were randomly divided into two study groups using the block randomization technique with a block size of 4 (1:1 ratio and four blocks). The group assigned to each participant was sequentially printed and placed in a similar opaque and sealed envelope by a third party who was not involved in the patients’ interview or evaluation. This person coded the drug containers based on provided sequence so that the participants were completely unaware of which group each number belonged to. Outcome assessors, randomizers, and statistical analysts were blind to the group allocation. Besides, the tablets and placebo were similar in size, shape, color, and odor.

### Intervention

Twenty-three CLBP patients were included in each study group. Both groups received pregabalin at a dose of 75 mg twice a day. Group B received agomelatine at a dose of 25 mg overnight. Patients of group A received a placebo instead of agomelatine. Patients’ evaluation was performed at weeks 0, 4, and 8. Liver enzymes were checked before the study and in the third week.

### Outcome measures

The major outcomes of this research were intensity of pain and impairment, as well as the prevalence of anxiety and depression. Brief Pain Inventory (BPI) interference scale was used to rate the level of pain.Moreover, the pain level was measured in seven daily activities, including general activity, walking, work, mood, enjoyment of life, relationships, and sleep. A score between 1 and 10 was assigned to each patient. Roland-Morris Disability Questionnaire (RMDQ) was used to assess CLBP-associated disability, a 24-item self-reported questionnaire with a score of 0–24. The higher the score, the greater the degree of impairment caused by CLBP. The Hospital Anxiety and Depression Scale (HADS) was used to assess anxiety and depression levels; its 14 items, each with a 0 to 3 point scale, provide a maximum score of 42. A higher score was indicative of more anxiety and depression. General health status was evaluated by the 36-Item Short Form Survey (SF-36), scored on a 0-100 scale, in which a higher score attributed to better general health status. General Health Questionnaire–28 (GHQ-28) evaluated the patients’ mental status, which identifies two main concerns: the inability to perform normal daily functions and the emergence of new and disturbing conditions. This questionnaire has 28 self-reported items (scores between 0 and 28), and a higher GHQ-28 score indicates higher levels of disorders. In all cases, Persian translations of the questionnaires were used, the validity and reliability of which had been proven in previous studies [14–18].

The secondary outcomes of the study were any adverse drug effects related to the combination of pregabalin and agomelatine of pregabalin alone.

### Sample size calculation and statistical analysis

The sample size was calculated using G-Power software. Considering the effect size of 0.75, the type I error of 5%, and the power of 80%, 23 patients in each group were found to be enough to perform this clinical trial.

SPSS for Windows version 16 (SPSS Inc., Chicago, Ill., USA) was used for statistical analysis. Descriptive data were presented as mean ± standard deviation or number and percentage. Kolmogorov-Smirnov test was used to determine if the data were normal. To compare the mean values across several groups, an Independent t-test or its non-parametric equivalent (Mann-Whitney U test) was performed. More than two means were compared using the ANOVA test. Repeated measure ANOVA was used to analyze the trend of variables over time. Regression models were used to control the confounders. Chi-squared test was used to compare qualitative data. A P-value less than 0.05 was considered significant.

## Results

Forty-six CLBP patients were randomly assigned to each study group. The mean age of the patients was 45.4 ± 12.9 years in the pregabalin ± placebo group and 44.1 ± 12 years in the pregabalin ± agomelatine group. This difference was not statistically significant (P = 0.71). The sex distribution differed significantly between two study groups, so the female gender was more frequent in the pregabalin ± placebo group (20 vs. 10, P = 0.001). Furthermore, the number of employed patients was significantly different between two study groups (4 vs.11, P = 0.013). No other significant difference was observed among the baseline characteristic features of the two study groups (Table 1).

**Table 1:** Comparison of the baseline characteristic features between the two study groups

Variable	Pregabalin ± Placebo (n = 23)	Pregabalin ± Agomelatine (n = 23)	P-value
**Sex** • **Male** • **Female**	3 (13) 20 (87)	13 (56.5) 10 (43.5)	0.001
**Age** • **< 30 years** • **30–40 years** • **40–50 years** • **50–60 years**	3 (13) 4 (17.4) 5 (21.70 11 (47.8)	5 (21.7) 4 (17.4) 2 (8.7) 12 (52.2)	0.89
**Disease duration** • **< 1 month** • **1–12 months** • **> 12 months**	1 (4.3) 8 (34.8) 14 (60.9)	2 (8.7) 6 (26.1) 15 (65.2)	0.91
**Education** • **Undergraduate** • **Bachelor** • **Master** • **Doctorate**	15 (65.2) 6 (26.1) 1 (4.3) 1 (4.3)	15 (65.2) 8 (34.8) 0 0	0.9
**Medicine taking** • **Regular** • **Irregular**	22 (95.7) 1 (4.3)	23 (100) 0	0.96
**Employment status** • **Employed** • **Unemployed**	4 (17.4) 19 (82.6)	11 (47.8) 12 (52.2)	0.013
**Marriage status** • **Single** • **Married**	4 (17.4) 19 (82.6)	5 (21.7) 18 (78.3)	0.88
**Body mass index** • **> 24.9 Kg/m**^**2**^ • **18.5–24.9 Kg/m**^**2**^ • **< 18.5Kg/m**^**2**^	13 (56.5) 9 (39.1) 1 (4.3)	14 (60.9) 8 (34.8) 1 (4.3)	0.93
**Smoking status** • **Smoker** • **Non-smoker**	3 (13) 20 (87)	4 (17.4) 19 (82.6)	0.95
**Physical activity** • **Yes** • **No**	21 (91.3) 4 (8.7)	21 (91.3) 2 (8.7)	-
Family history of CLBP* • **Yes** • **No**	13 (56.5) 10 (43.5)	15 (65.2) 8 (34.8)	0.79

*CLBP: Chronic low back pain

Data are presented as mean ± SD or number (%). A P < 0.05 is considered significant

In group A (pregabalin + placebo group), all outcome measures showed significant improvement during weeks 4 and 8 compared to week 0. The same improvements were observed in group B (pregabalin + agomelatine group) (Tables 2 and 3).

**Table 2:** before-after comparison of the outcome measures in patient receiving pregabalin ± placebo

Outcome of interest	Pregabalin ± Placebo (n = 23)	F	P-Value
**BPI** • **Week 0** • **Week 4** • **Week 8**	5.15 ± 1.96 4.39 ± 1.66 3.41 ± 1.71	46.77	< 0.001
**RMDQ** • **Week 0** • **Week 4** • **Week 8**	10.83 ± 5.91 8.78 ± 4.69 6.7 ± 3.32	25.72	< 0.001
**SF-36** • **Week 0** • **Week 4** • **Week 8**	47.52 ± 16.94 53.52 ± 14.61 59.13 ± 11.62	34.28	< 0.001
**HADS** • **Week 0** • **Week 4** • **Week 8**	13.22 ± 5.7 11.48 ± 4.35 10.74 ± 4.59	52.33	0.007
**GHQ-28** • **Week 0** • **Week 4** • **Week 8**	27.96 ± 10.8 25.17 ± 8.95 21.26 ± 6.62	19.14	< 0.001

BPI: Brief Pain Inventory; RMDQ: Roland-Morris Disability Questionnaire; SF-36: 36-Item Short Form Survey; HADS: Hospital Anxiety and Depression Scale; GHQ-28: General Health Questionnaire *–* 28

Data are presented with mean ± SD and analyzed by repeated measure ANOVA and Greenhouse-Geisser correction. A P < 0.05 is considered significant

**Table 3:** Before-after comparison of the outcome measures in patient receiving pregabalin ± agomelatine

Outcome of interest	Pregabalin ± Agomelatine (n = 23)	F	P-Value
**BPI** • **Week 0** • **Week 4** • **Week 8**	5.72 ± 1.25 4.21 ± 0.8 3.23 ± 0.97	67.63	< 0.001
**RMDQ** • **Week 0** • **Week 4** • **Week 8**	10.7 ± 4.94 7.65 ± 4.16 5.87 ± 3.82	43.03	< 0.001
**SF-36** • **Week 0** • **Week 4** • **Week 8**	46.78 ± 13.65 56.43 ± 9.47 62.61 ± 9.99	49.55	< 0.001
**HADS** • **Week 0** • **Week 4** • **Week 8**	14.52 ± 6.02 11.61 ± 4.57 9.74 ± 5.24	14.1	< 0.001
**GHQ-28** • **Week 0** • **Week 4** • **Week 8**	28.52 ± 8.58 21 ± 7.53 15.43 ± 5.8	90.36	< 0.001

BPI: Brief Pain Inventory; RMDQ: Roland-Morris Disability Questionnaire; SF-36: 36-Item Short Form Survey; HADS: Hospital Anxiety and Depression Scale; GHQ-28: General Health Questionnaire *–* 28

Data are presented with mean ± SD and analyzed by repeated measure ANOVA and Greenhouse-Geisser correction. A P < 0.05 is considered significant

**Fig. 1:**
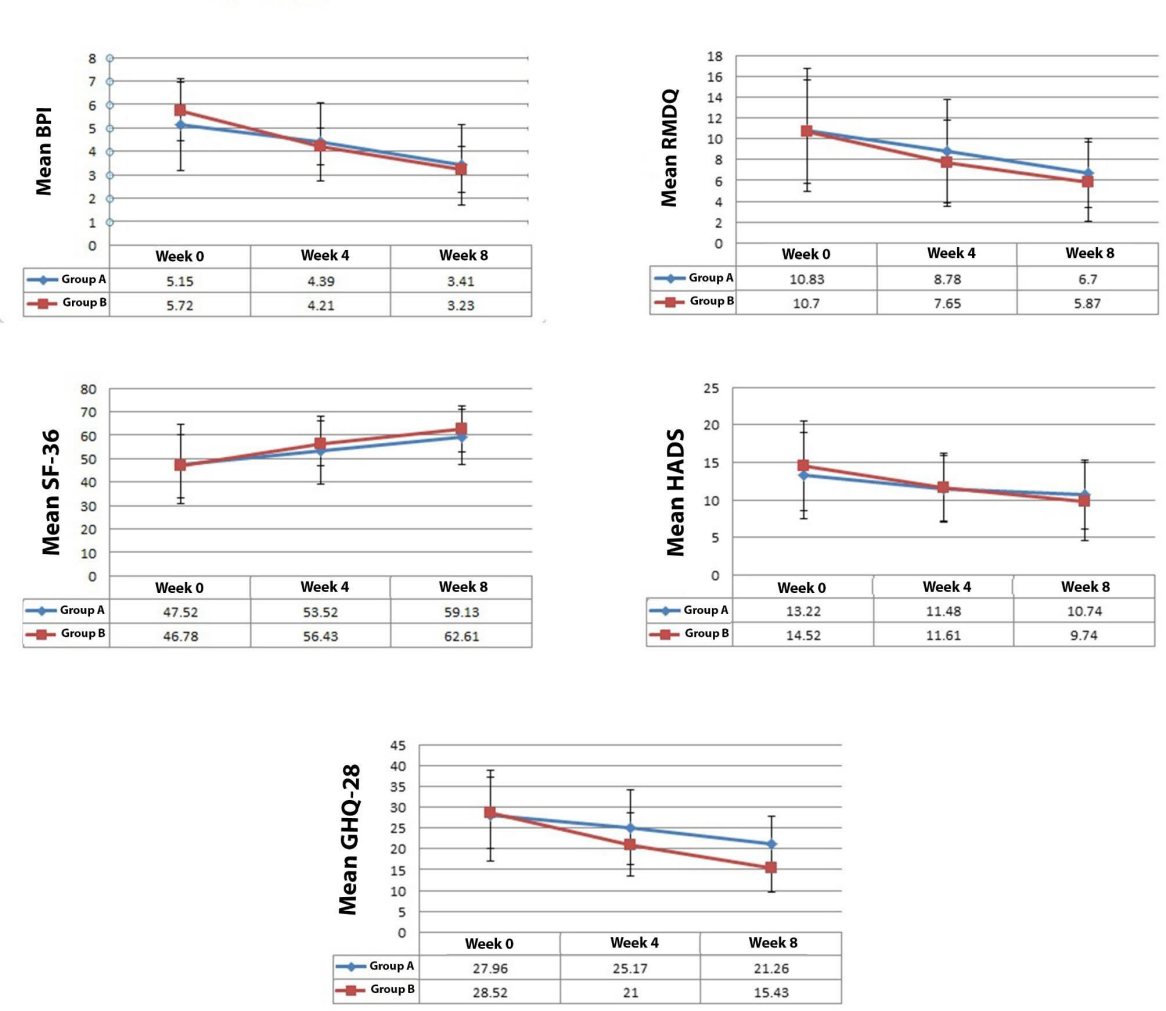
Comparison of the mean outcome measures over the time between the group A (pregabalin & placebo) and group B (pregabalin and agomelatine). (BPI: Brief Pain Inventory; RMDQ: Roland-Morris Disability Questionnaire; SF-36: 36-Item Short Form *Survey; HADS*: Hospital Anxiety and Depression Scale; GHQ-28: *General Health Questionnaire – 28.)*

The mean improvement of BPI was not significantly different between two study groups (P = 0.61). The mean improvement of RMDQ was not significantly different between two study groups (P = 0.44). Furthermore, the mean improvement of SF-36 and HADS was not statistically different between two study groups (P = 0.28 and P = 0.49, respectively). The mean improvement of GHQ-28 was significantly higher in group B (P = 0.003) (Table 4; Fig. 1).

**Table 4:** Comparison of the improvement of outcome measures between the two study groups

Outcome of interest	Pregabalin ± Placebo (n = 23)	Pregabalin ± agomelatine (n = 23)	t	P-Value
**BPI**	1.74 ± 0.96	2.49 ± 1.05	0.502	0.61
**RMDQ**	4.13 ± 1.87	4.83 ± 1.89	0.781	0.44
**SF-36**	11.61 ± 4.28	15.83 ± 4.96	0.282	0.28
**HADS**	2.48 ± 4.2	4.78 ± 5.4	0.495	0.49
**GHQ-28**	3.7 ± 1.22	13.1 ± 4.71	3.173	0.003

BPI: Brief Pain Inventory; RMDQ: Roland-Morris Disability Questionnaire; SF-36: 36-Item Short Form Survey; HADS: Hospital Anxiety and Depression Scale; GHQ-28: General Health Questionnaire *–* 28

Data are presented with mean ± SD and analyzed by paired t-test. A P < 0.05 is considered significant

## Adverse effects

None of the patients in either of the two trial groups had serious side effects that required therapy to be stopped. Patients in both groups regularly had mild side effects as nausea, headaches, trouble sleeping, lightheadedness, palpitations, diarrhea, and muscular discomfort. There were no statistically significant differences in the distribution of minor side effects between the two research groups (Table 5).

**Table 5:** Comparison of minor adverse effects between the two study groups

Adverse effect	Pregabalin ± Placebo (n = 23)	Pregabalin ± agomelatine (n = 23)	P-Value
**Nausea **	5 (21.7)	6 (26)	0.73
**Headache**	2 (8.7)	3 (13)	0.63
**Sleep disorder**	6 (26)	9 (39)	0.34
**Lightheadedness**	3 (13)	3 (13)	-
**Palpitation**	2 (8.7)	2 (8.7)	-
**Diarrhea**	1 (4.3)	1 (4.3)	-
**Muscle pain**	2 (8.7)	3 (13)	0.63

Data are demonstrated as number (%). A P < 0.05 is considered significant

## Discussion

We investigated the therapeutic effect of co-administration of pregabalin and agomelatine versus pregabalin alone to treat CLBP. Both treatments significantly improved the patients’ general and mental health status of pain, disability, anxiety, and depression. Improvement of mental health was significantly more prominent in the pregabalin + agomelatine group. No other significant difference was found among the outcome measures of the two study groups. The addition of agomelatine to the treatment of CLBP was well tolerated and not associated with any specific side effects.

Previous studies have shown that melatonin has analgesic characteristics that may be used to treat chronic pain disorders such fibromyalgia, headaches, chronic back pain, and rheumatoid arthritis [19].Considering the agomelatine as a synthetic melatonin agonist, the same analgesic properties could be provided by agomelatine administration. The agomelatine significantly improved depression, anxiety, and pain in fibromyalgia patients [20, 21]. However, the analgesic effect of agomelatine on CLBP has not been investigated in an earlier study.

Chenaf et al. used three rat models of neuropathic pain (toxic, metabolic, and traumatic) etiology to investigate the effect of melatonergic, 5-HT2C, α-2, and β-1/2 adrenergic receptor antagonists on the antihypersensitivity effect of agomelatine. In the metabolic and traumatic models, a single dose of agomelatine dramatically and dose-dependently decreased mechanical sensitivity. This effect was verified in the traumatic model after two weeks of daily dosing, and agomelatine also showed a significant antihypersensitivity impact in the toxic model.The antihypersensitivity effect of agomelatine included melatonergic adrenergic receptors, 5-HT2C and α-2. But, it did not involve beta-adrenergic receptors. Isobologic analysis showed that the combination of agomelatine and gabapentin has additive effects. Based on the results of this study, the agomelatine contained a noticeable antihypersensitivity impact in three models of neuropathic pain, which is exerted by melatonergic receptors and 5-HT2C, and α-2 adrenergic receptors [10]. The study of Aydın et al. revealed the curative effect of agomelatine on painful diabetic neuropathy in rats and suggested that these effects are mediated by rising synaptic catecholamine levels and through interactions with both α- and β-adrenoceptors [22]. The study of M’Dahoma et al. revealed that co-administration of agomelatine and gabapentin is a potent anti-allodynic combination in neuropathic rats with the ligated infraorbital or sciatic nerve, which is medicated by α2- and β2-adrenoreceptor noradrenergic neurotransmission [23]. Considering neuropathic pain is a crucial component of CLBP, agomelatine might possibly alleviate CLBP symptoms. The mental condition of patients who got agomelatine plus pregabalin improved more than those who received pregabalin alone, confirming this result.Recent studies suggest that agomelatine has analgesic effects against nociceptive pain. Kivrak et al., in an experimental study, evaluated the effect of agomelatine on the nociceptive system. They randomized 24 male Swiss albino mice into three study groups: Group A (12.5 mg/kg of agomelatine), Group B (25 mg/kg agomelatine), and Group C (physiological saline via intraperitoneal injection). Agomelatine’s nociceptive effects were evaluated using the hot plate technique, and pain threshold values were noted in the 30th and 60th-minute results. The agomelatine groups had larger pain thresholds than the control group. In comparison to the other research groups, group B’s pain threshold was greater after 30 min [24].The study of Ozcan et al. revealed that agomelatine could augment the anti-nociceptive effects of morphine in the mice model of diabetic neuropathy [25]. The potential of anti-nociceptive effects of agomelatine, besides its anti-neuropathic effects, further support its implication to treat CLBP.

There is no clear consensus on pregabalin’s effectiveness to treat CLBP. While some studies showed significant improvement in CLBP symptoms following pregabalin administration [26–30], others have found no clear benefit for pregabalin administration for CLBP treatment. Our study revealed significant improvement in CLBP symptoms in the patients who received pregabalin alone. This observed discrepancy among the studies could be attributed to the patients’ composition regarding the components of pain. Cumulative evidence does not support the administration of pregabalin in the patients with a neuropathic part of CLBP pain [31, 32]. However, this hypothesis should be examined in future studies because the components of pain were investigated neither in our study nor in previous studies.

The present study results reveal that co-administration of pregabalin and agomelatine provides additive value to treat CLBP. This benefit is well presented in the mental status of the patients.

This study has certain restrictions. The study’s key flaw was the sparse patient population, which would have significantly impacted the validity of the statistical analysis. Due to the limited patient population, there were also notable disparities between the two research groups’ fundamental characteristics, such sex and work position.Therefore, future complementary studies with larger patient numbers are required to confirm the results of the present study.

This study’s components of pain (nociceptive and neuropathic) were not differentiated. Therefore, we do not know how many patients have had neuropathic pain. Knowing this will help the personalized use of agomelatine to treat chronic back pain. Therefore, we suggest evaluation of the pain components of patients in future studies before assessing of agomelatine effect.

We used GHQ-28 Questionnaire to evaluate the patients’ mental status. However, it is more a screening test than a diagnostic test. Evaluation with Structured Clinical Interview for DSM Disorders (SCID) or DSM–5-TR would better represent the mental status of the patients and could be considered in future investigations.

Only clinical observation was included in the study’s side effects questionnaire. To better address the side effects of the treatments, paraclinical assessments such as the measurement of liver enzymes in blood samples might be undertaken in addition to the clinical assessment. Finally, the present study lacks a placebo group to remove the effect of placebo-related improvement and attribute the general health improvement to pregabalin alone. Therefore, the inclusion of a placebo group is recommended for future projects in this field.

## Conclusion

Co-administration of pregabalin and agomelatin is more effective in treating CLBP symptoms than pregabalin plus placebo. This additive value was pronounced in the mental status of CLBP patients. Future studies with more significant patient numbers should better characterize the agomelatine analgesic effects in CLBP patients.

## Data Availability

The datasets used and/or analysed during the current study are available from the corresponding author on reasonable request.
